# Research on Rehabilitation Training Strategies Using Multimodal Virtual Scene Stimulation

**DOI:** 10.3389/fnagi.2022.892178

**Published:** 2022-06-30

**Authors:** Ping Xie, Zihao Wang, Zengyong Li, Ying Wang, Nianwen Wang, Zhenhu Liang, Juan Wang, Xiaoling Chen

**Affiliations:** ^1^Key Laboratory of Measurement Technology and Instrumentation of Hebei Province, Institute of Electric Engineering, Yanshan University, Qinhuangdao, China; ^2^National Research Center for Rehabilitation Technical Aids, Beijing, China

**Keywords:** brain-computer interface, motor imagery, virtual reality, neural activation, virtual rehabilitation

## Abstract

It is difficult for stroke patients with flaccid paralysis to receive passive rehabilitation training. Therefore, virtual rehabilitation technology that integrates the motor imagery brain-computer interface and virtual reality technology has been applied to the field of stroke rehabilitation and has evolved into a physical rehabilitation training method. This virtual rehabilitation technology can enhance the initiative and adaptability of patient rehabilitation. To maximize the deep activation of the subjects motor nerves and accelerate the remodeling mechanism of motor nerve function, this study designed a brain-computer interface rehabilitation training strategy using different virtual scenes, including static scenes, dynamic scenes, and VR scenes. Including static scenes, dynamic scenes, and VR scenes. We compared and analyzed the degree of neural activation and the recognition rate of motor imagery in stroke patients after motor imagery training using stimulation of different virtual scenes, The results show that under the three scenarios, The order of degree of neural activation and the recognition rate of motor imagery from high to low is: VR scenes, dynamic scenes, static scenes. This paper provided the research basis for a virtual rehabilitation strategy that could integrate the motor imagery brain-computer interface and virtual reality technology.

## Introduction

The brain-computer interface (BCI) is a direct communication and control channel established between the human brain and computer or other electronic devices (Wolpaw et al., 2002). Through this channel, people can express ideas or manipulate equipment directly through the brain without any language or action, which can effectively enhance the ability of patients with severe physical disabilities to communicate with the outside world or to control the external environment to improve the quality of life of patients (Ren et al., 2004). Virtual reality (VR) technology creates a virtual world through the use of a new computer system, allowing the experiencer to integrate into the virtual environment and achieve mutual interaction. In addition to being used in the gaming industry, VR also can help stroke patients with flaccid paralysis perform purposeful training in a virtual environment, thereby improving the effect of rehabilitation training. Motor imagery (MI) is defined as the cognitive activity in which a subject imagines a movement without actually performing the movement (Vries and Mulder, 2007), and it is a common application paradigm in the field of brain-computer interface research (Mattia et al., 2012). This method realizes communication and control with external devices by imaging body movements (Xu et al., 2013).

Motor imagery brain-computer interface (MI-BCI) is a type of BCI that recognizes the patient’s motor imagery intention by guiding the patient to perform motor imagery based on motor imagery therapy (Clark et al., 2019). It can be used to effectively remodel the central nervous system in patients with motor dysfunction (Guger et al., 2017). VR technology can provide patients with a more immersive training environment (Ren et al., 2020), help patients perform motor imagery more accurately (Bayliss and Ballard, 2000), and generate more easily identifiable electroencephalography (EEG) signals (Li et al., 2018). Remsik et al. (2016) reviewed 17 independent MI-BCI stroke rehabilitation studies, and they found that 16 produced significant treatment effects. Studies have shown that both motor imagery and actual movement can activate bilateral premotor areas (Pfurtscheller and Neuper, 2001), parietal lobes, basal ganglia, and cerebellum. Studies (Pfurtscheller and Neuper, 1997) also have shown that stroke patients can perform motor imagery to partially activate the damaged motor network (Fumanal-Idocin et al., 2021). In addition, studies have found that the rehabilitation treatment model combining BCI and VR technology is suitable for people suffering from stroke (Varsehi and Firoozabadi, 2021), depression, addiction and other diseases (Takenaka et al., 2021). VR-guided action offers the advantages of intuitive and specific actions and a strong sense of substitution (Velasquez-Martinez et al., 2020). Well-designed scene feedback can produce neural activation (Vourvopoulos et al., 2015).

Rehabilitation training strategies based on VR and MI-BCI have the following limitations (Benitez-Andonegui et al., 2020). First, the current rehabilitation strategy based on MI-BCI improves accuracy primarily by improving the algorithm without using a scene stimulation to improve the neural activation of the subject (Xiao and Fang, 2021), and thereby improving the quality of the EEG signals to improve accuracy (Bagarinao et al., 2020). Second, the virtual rehabilitation scene is singular (Vidaurre et al., 2020), the individual adaptability is poor, and few studies (Li et al., 2021) have compared the neural region activation and enhanced EEG signals feature mechanisms in different scenes (Barsotti et al., 2015). Third, most of the existing virtual rehabilitation training strategies are performed by observing virtual scenes on a computer screen (Moctezuma and Molinas, 2020). This training mode is not only less immersive, but also easily disturbed by the external environment, which introduces difficulties to the rehabilitation training (Gomez-Pilar et al., 2016). Finally, visual feedback training is lacking during rehabilitation. At the neural mechanism level, visual feedback training promotes brain plasticity changes and functional reorganization through the activation of the mirror neuron system, thereby promoting the recovery of motor function (Birbaumer et al., 2006).

In this study, to examine ways to improve the deep activation mechanism of motor nerves in stroke patients with flaccid paralysis, we designed different virtual scenes and training tasks to stimulate motor imagery from different angles. The virtual scenes include the following: limb MI in static scenes, such as text and pictures; limb MI in dynamic scenes of three-dimensional (3D) life and virtual games; and limb MI in the VR environment. This study compared and analyzed energy changes in the motor areas of the brain and the recognition rate of motor imagery before and after rehabilitation training using three-scene stimulation. This study verified the positive activation effect of rehabilitation training strategy on stroke patients and explored the brain activation mechanism using stimulation with different virtual scenes.

## Materials and Methods

### Experimental Paradigm Under Virtual Scenes Stimulation

#### Design of Virtual Scenes and Training Tasks

**FIGURE 1:**
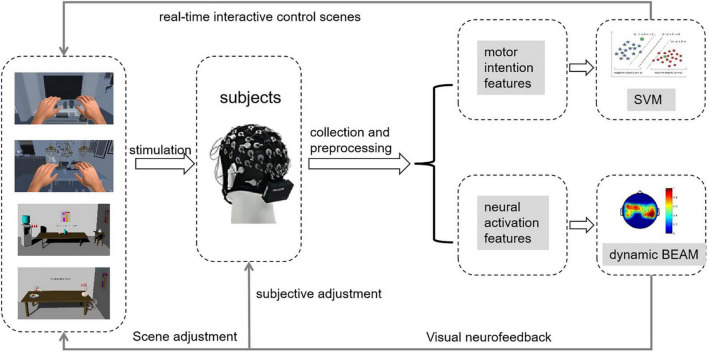
Virtual rehabilitation training strategy map.

As shown in Figure 1, in this study, we designed a rehabilitation training strategy based on MI-BCI and VR. Multiple-evoked MI stimulation scenes (e.g., static scenes, dynamic scenes, and VR scenes) induced subjects to perform limb motor imagery. The EEG signals of the brain motor area were collected in real time and then the signals were subjected to preprocessing, intention feature extraction, and intention recognition. Finally, the results were output to the scene for interactive control.

The virtual scenes were interesting and immersive, and the patients had a strong awareness of active training. In this study, we first built the scenes. Then, we added the image of the virtual left and right hands into the scenes and set the training tasks and audio-visual feedback. Finally, through human-computer interaction control, the subjects could feel that they were performing actual body movements. Then we guided the subjects to perform active limb motor imagery.

In addition, we observed the brain electrical activity mapping of the subject and analyzed the degree of neural activation during the subject’s brain electrical activity mapping. We observed the activation changes in the brain areas through brain electrical activity mapping and analyzed the effect of training on the subjects. According to the current neural activity in the motor area of the brain, we adaptively adjusted the training scenes to ensure that the subjects could continuously achieve maximum activation of nerves and to accelerate the remodeling of nerve functions.

Different research protocols can lead to different promotion effects of the BCI rehabilitation training system on either the unaffected or affected side of the cerebral hemisphere (Dodd et al., 2017). Different subjects respond differently to different scenes, and the activation areas and intensity of the cerebral cortex and the EEG signal features also are different when the same subject performs different tasks. Considering these differences, this study used the Unity 3D platform to design the training scenes (static and dynamic scenes) in the computer screen as well as the training scenes in the VR environment. We also designed some life-skills training in the scene to feature rehabilitation tasks (e.g., holding goods, pouring water, and picking food).

**FIGURE 2:**
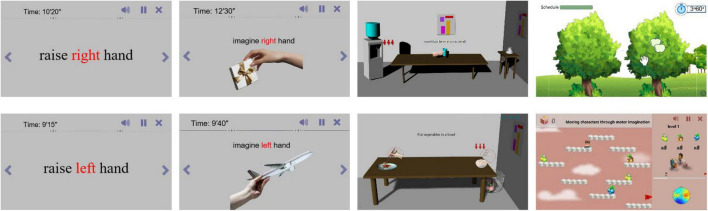
Static and dynamic scenes without VR.

As shown in Figure 2, this study designed four different static and dynamic scenes without using VR. They included static text scenes, static picture scenes, dynamic 3D life scenes, and dynamic virtual game scenes.

**FIGURE 3:**
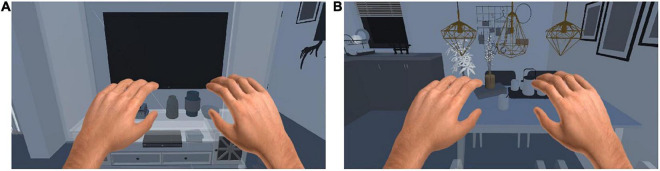
Training scenes in VR enviroment. **(A)** One scene for computer. **(B)** One scence for dinner.

As shown in Figure 3, we also designed scenes in the VR environment to enhance the immersion and realism of the subject’s training in this study. The control method of the training tasks required the patient to perform motor imagery according to the prompt. In other words, when the patient’s use of left (right) motor imagery was recognized, the corresponding limb in the scenes would perform the corresponding movement.

The following describes the scene in detail.

Static scene: Mainly contains text, pictures, etc., the stimulation effect is poor, but the stimulation is more direct. Directly prompt the subjects to perform motor imagery through left, right, etc.

Dynamic scenes: Mainly include videos, games, etc., with general stimulation effects and strong interactivity. Realize motor imagery by playing videos or playing games.

VR scene: The main stimulation mode is the same as that under the computer monitor, but after VR rendering, the sense of immersion is strong, making the patient feel immersive, and the stimulation effect is the best.

#### Experimental Design

##### Experimental Principle and Process

The purpose of this study was to verify the effect of the rehabilitation training strategy to improve the subjects’ limb control ability during motor imagery. At the same time, we studied the differences in brain activation when subjects were stimulated to perform motor imagery in different scenes. This enabled us to analyze the effect of neural activation when subjects performed motor imagery using stimulation with different virtual scenes.

**FIGURE 4:**
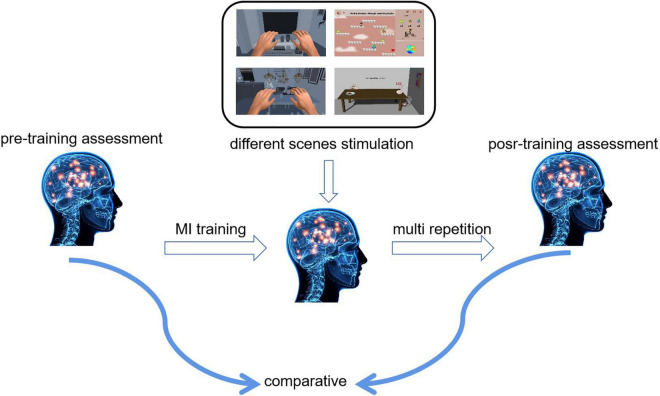
Experimental schematics.

In this study, we designed a controlled experiment to compare and analyze the changes in neural activity of the brain and to determine the recognition rate of motor imagery using stimulations with different virtual scenes to find the mechanism of neural deep activation. Figure 4 shows a schematic diagram of the experiment. We assessed the subjects’ motor imagery ability before training and then subjects performed multiple motor imagery training. After completing the training, participants finished a post-training assessment.

We selected nine healthy college students in good mental condition as subjects (all male; average age: 24 ± 2 years old). All subjects are right handedness. The experiment required that all the subjects had not completed similar experiments before and had no history of neurological diseases. All the subjects were informed of the research intention of the trial, the details of the study, and the potential dangers associated with the experiment. Additionally, all of the experiments were conducted within 3 h of the subject having eaten at noon, and each subject had to close his eyes and rest for 5 min before starting motor imagery. Doing so could relieve tension and anxiety and ensured that subjects were in a good mental state.

We grouped the nine subjects equally into three groups: static scene control group, called S1–S3; dynamic scene experimental group, called S4–S6; and VR scene experimental group, called S7–S9. Except for the different virtual scenes for stimulating motor imagery training, all of the other conditions were the same. All the subjects performed a total of 17 days of experiments (14 days of motor imagery task training experiments and 3 days of motor imagery assessment experiments). The specific experimental process was as follows: Subjects completed three sets of enhanced motor imagery paradigm training per day, and each group included 40 trials. In other words, subjects had to complete a total of 120 limb motor imagery trials per day. In the 14-day motor imagery training using virtual scene stimulation, we obtained the correct rate of 1,680 limb motor images for each subject.

Subjects also performed three motor imagery assessment experiments. Three experiments were conducted on the day before the motor imagery training, the day after the training was completed for 7 days, and the day after the training was completed for 14 days. The subjects completed three sets of motor imagery tasks according to the assessment paradigm, and each group contained 30 motor imagery trials. We collected 19 channels of EEG data during the trials and obtained a total of 270 EEG data points for each subject in the motor imagery trials.

##### Experimental Paradigm

**FIGURE 5:**
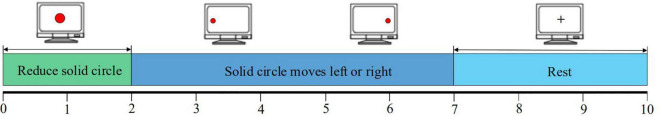
Assessment paradigm before and after enhanced MI training.

Figure 5 shows the experimental paradigm for the pre-assessment and post-assessment in the enhanced motor imagery training. A single trial contained three periods totaling 10 s. From 0 to 2 s, a red dot appeared in the center of the screen and then shrunk to remind the subjects to concentrate to start the next motor imagery. From 2 to 7 s, the subjects had to focus on the direction of the left/right movement of the red dot on the screen and performed left/right hand (or left/right limb) motor imagery. From 7 to 10 s, a plus sign (+) appeared in the center of the screen to remind the subjects that this trial had ended. During this period, subjects would rest for 3 s and then they would enter the next trial to repeat this experimental process.

**FIGURE 6:**
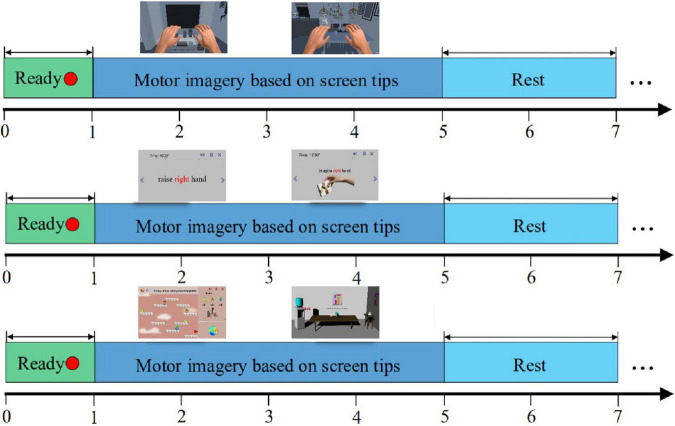
Experimental paradigm of enhanced MI training in different scenes.

The experimental paradigm of enhanced motor imagery training in different scenes is shown in Figure 6. We divided the training process into three groups: the enhanced static scenes control group, the enhanced dynamic scenes experimental group, and the VR scenes experimental group. The motor imagery training process adopted an interactive mode of real-time motor imagery feedback. A single trial contained three periods totaling 10 s. From 0 to 1 s, a red dot appeared in the center of the screen to remind the subjects to concentrate on the start of the motor imagery. At the end of 1 s, the red dot disappeared, which reminded the subjects to start the limb motor imagery. From 1 to 5 s, subjects had to focus on the prompt information provided on the screen and performed left/right hand (or left/right limb) motor imagery. Different prompt information was given to different groups. From 5 to 7 s, the results of the classification model in the motor imagery appeared on the screen, and the virtual characters and limbs in the scenes were controlled to move accordingly. During this period, subjects took a short rest until the end of 7 s. Then, the subjects would enter the next trial and repeat this experimental process.

### Electroencephalography Signals Acquisition and Preprocessing

The electrode distribution of EEG data acquisition adopted the international standard 10- to 20-electrode lead positioning. We set the reference electrode in the central area at the top of the head and set the sampling frequency to 1,000 Hz. The acquisition channels included 19 channels in the motor nerve-related area, namely, FC1, FC3, FC5, C1, C3, C5, CP1, CP3, CP5, Cz, FC2, FC4, FC6, C2, C4, C6, CP2, CP4, and CP6.

Most of the EEG acquisition devices were non-invasive dry electrodes. Although EEG acquisition was simpler and more convenient, it was unavoidable that various noises would appear in the acquisition process, such as EOG, ECG, and EMG. This seriously interfered with the subsequent analysis of EEG signals and affected the results of EEG signal analysis. Therefore, it was necessary to preprocess the collected raw EEG signals to remove noises.

The main noises included baseline drift caused by the device, 50 Hz power frequency interference, and EOG artifacts formed by blinking. We used the moving average method to remove baseline drift and used an adaptive 50 Hz filter to remove power frequency interference. We used an independent component analysis to remove EOG artifacts and used the sixth-order Butterworth filter as the band-pass filter. Finally, we acquired 8–32 Hz EEG signals.

## Results

### Analysis of Energy Changes in Brain Motor Areas

#### Brain Electrical Activity Mapping

Studies have shown that when subjects perform motor imagery, the energy features of EEG signals in the motor areas of the brain must change. In this study, we used brain electrical activity mapping to analyze the neural activation of multiple channels in the motor area, and visual neurofeedback was provided.

Brain electrical activity mapping (BEAM) is a commonly used method for multichannel EEG signal analysis. This method collects EEG signals from multiple channels of the head, performs fourth-order energy extraction, and then uses color bands and digitization to represent different gray levels. Finally, a dynamic BEAM can be drawn.

#### Brain Motor Area Energy Analysis Based on Brain Electrical Activity Mapping

**FIGURE 7:**
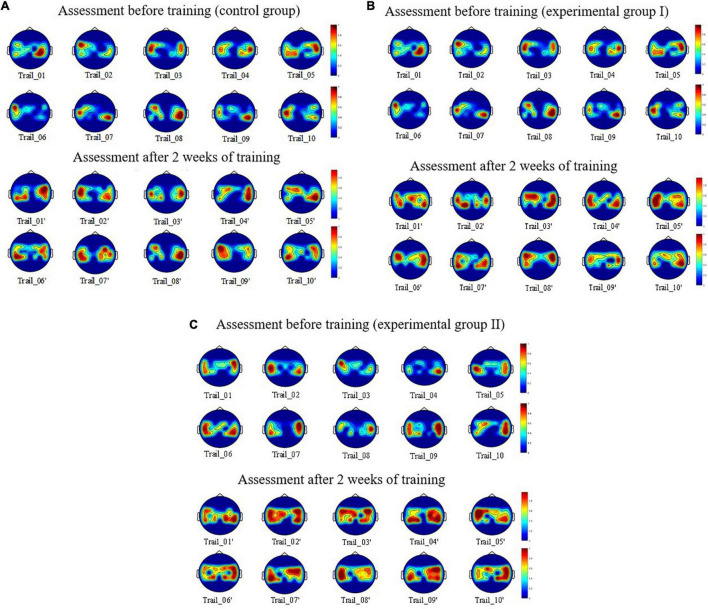
Spatial distribution of the brain activation between the before and after enhanced MI training. **(A)** For control group. **(B)** For experimental group. **(C)** For experimental group II.

On the basis of these experiments, we obtained the EEG data of nine subjects when they conducted motor imagery assessment on the day before the motor imagery training and on the day after the training had been completed for 14 days. We obtained and normalized fourth-order cumulative energy value of each channel for a single trial for each subject. Then, we used the energy value of each channel for multiple trials of nine subjects, which then was used as the input parameter for drawing the BEAM. We obtained the spatial distribution map of the neural activation in different lead positions of the left and right limb motor imagery, as shown in Figure 7.

Figure 7 shows the spatial distribution of neural activation in the brain regions of the subjects: S1, S4, and S8 before and after 10 trials of enhanced motor imagery training. Figure 7A shows that after the subjects were trained in the rehabilitation strategy designed in this study, the activation range of motor nerves in the brain region was significantly expanded (i.e., the activation breadth of motor imagery nerves increased). Figure 7B shows that after the subjects were trained in the rehabilitation strategy, the color of the activation area was darker (i.e., the ERD/ERS phenomenon was more obvious), which indicated that the activation depth of the motor imagery nerves increased. Figure 7C shows that compared with static scenes and dynamic scenes, this strategy had a more obvious effect on the activation breadth and depth of motor imagery nerves in VR scenes.

### Electroencephalography Signals Feature Extraction, the Modeling of Electroencephalography Signals, and Analysis of the Recognition Rate of Motor Imagery

#### Electroencephalography Signal Feature Extraction

Feature extraction is the mathematical transformation or mapping of the input signal to obtain eigenvalues that are easier to observe and monitor. The widely used EEG signals that feature the extraction methods used during motor imagery mainly include the following: power spectrum estimation method, wavelet transform, independent component analysis, and common spatial pattern (Xin and Wang, 2017). In this study, we selected three feature combinations of mean square error, power spectral density, and common spatial pattern from the time domain, frequency domain, and spatial domain of EEG signal as the feature quantities for motor imagery intention recognition (Yang et al., 2012).

Furthermore, we used the existing particle swarm optimization (PSO)-support vector machine (SVM) as a classifier to perform motion imagery intention recognition.

#### Analysis of the Recognition Rate of Motor Imagery

Studies have shown that there is a positive correlation between the degree of active participation, the activity degree of motor nerves, the performance of classification models, and the recognition rate of motor imagery when subjects perform motor imagery. The higher the recognition rate of motor imagery, the higher the active participation of the subjects in motor imagery and the stronger the control ability of motor nerves. The recognition rate of motor imagery can be used as an indicator to stimulate motor imagery ability in different scenes. In this study, the analysis of the recognition rate of motor imagery intention included the following two aspects: online MI training and offline MI assessment.

##### Online Motor Imagery Training Recognition Rate Analysis

We counted the recognition rate of the nine subjects who completed online MI training in virtual scenes within 14 days (Table 1). The recognition rate was the online recognition result obtained statistically using the experimental paradigm designed in this study. The feature quantities used in the classification model were mean square error, power spectral density, and common spatial pattern features. The classification model was PSO-SVM.

**TABLE 1:** Statistical results of online recognition rate during MI training in different virtual scenes (%).

Group	Static scenes (control group)			Dynamic scenes (experimental group I)			VR scenes (experimental group II)		
Training day	S1	S2	S3	S4	S5	S6	S7	S8	S9
Day_01	60.9%	55.9%	57.6%	61.8%	58.5%	62.6%	62.6%	59.4%	60.3%
Day_02	65.7%	57.4%	60.7%	60.7%	64.8%	59.1%	64.1%	63.1%	64.7%
Day_03	63.4%	61.6%	65.2%	65.1%	67.4%	67.4%	67.4%	64.8%	71.1%
Day_04	69.1%	59.3%	61.5%	69.1%	59.3%	65.9%	71.8%	71.0%	73.9%
Day_05	67.4%	62.7%	70.7%	72.4%	62.6%	70.7%	65.7%	67.3%	74.5%
Day_06	65.9%	66.5%	65.9%	76.8%	65.7%	72.6%	76.8%	73.5%	72.2%
Day_07	56.5%	65.2%	67.6%	70.7%	70.7%	70.7%	72.6%	74.0%	70.7%
Day_08	67.7%	69.8%	56.6%	69.3%	69.3%	65.1%	73.2%	72.7%	75.9%
Day_09	69.3%	67.3%	63.5%	78.2%*	74.3%	74.3%	79.3%	79.2%*	80.8%
Day_10	72.4%	72.7%*	65.6%	72.6%	77.4%*	67.4%	76.6%	76.7%	74.2%
Day_11	70.1%	62.1%	69.3%	67.6%	69.1%	70.0%	82.4%*	72.1%	79.5%
Day_12	74.9%*	69.3%	70.7%	76.6%	68.4%	67.5%	73.4%	70.4%	83.0%
Day_13	68.2%	67.1%	71.6%*	72.5%	70.0%	73.5%	78.3%	76.8%	75.3%
Day_14	70.9%	67.6%	67.6%	74.2%	71.7%	74.0%*	80.0%	78.2%	88.8%*
Average recognition rate	67.3%	64.6%	65.3%	70.5%	67.8%	68.6%	73.2%	71.4%	74.6%
Standard deviation	0.045	0.042	0.046	0.052	0.051	0.043	0.060	0.057	0.070

*denotes the highest MI recognition rate for each subject across the 14 days.

Each subject performed 120 motor imagery trials per day. We counted the number of correct motor imagery classifications per day and calculated each subject’s single-day recognition rate, average recognition rate, and standard deviation within 14 days. The data marked with an asterisk (*) in the table indicate that the subject had the highest recognition rate on this day.

According to Table 1, by completing the enhanced motor imagery training task, the subjects’ motor imagery recognition rates improved to varying degrees. After training, the average recognition rate of motor imagery for three subjects (S1–S3) in static scenes (control group) was between 64.5 and 67.5%, the average recognition rate of motor imagery for three subjects (S4–S6) in dynamic scenes (experimental group I) was between 67.5 and 70.5%, and the average recognition rate of motor imagery for three subjects (S7–S9) in VR scenes (experimental group II) was between 71.0 and 75.0%.

These data showed that the rehabilitation training strategy designed in this study could improve subjects’ limb motor imagery ability. The subjects’ motor imagination ability in these three scenarios is from weak to strong: static scene, dynamic scene, and VR scene.

##### Offline Motor Imagery Assessment Recognition Rate Analysis

By analyzing the improvement range of the subjects’ motor imagery recognition rate under different training periods, we used an assessment method to evaluate whether the rehabilitation training strategy could improve the subjects’ motor imagery ability. We collected the EEG data of nine subjects when they conducted motor imagery assessments 1 day before the motor imagery training, the day after the training was completed for 7 days, and the day after the training was completed for 14 days. Each subject completed three sets of motor imagery tasks per day, and each set consisted of 30 random left and right hand (limb) motor imagery trials.

We subjected the collected data to offline recognition rate analysis. To obtain accurate results, we used a three-fold cross-validation method, as follows: First, we divided the 90 motor imagery datasets into three groups on average. One group was taken out each time as the test data, and the remaining data were used as the training data to complete the PSO-SVM model training. Then, we used the trained model to classify the test data. Finally, we averaged the obtained three groups of classification results as the final recognition rate. The statistical results are given in Table 2.

**TABLE 2:** Offline evaluation of recognition rate on different virtual scenes before, during and after enhanced MI training (%).

Group	Subjects	Recognition rate before the training	Recognition rate after 7-day training	Increasing range (%)	Recognition rate after 14-day training	Increasing range (%)
Static scenes	S1	63.6%	69.7%	6.1	72.8%	3.1
	S2	61.6%	66.1%	4.5	68.9%	2.8
	S3	64.1%	68.4%	4.3	72.1%	3.7
Dynamic scenes	S4	63.8%	69.6%	5.8	74.7%	5.1
	S5	64.1%	71.4%	7.3	76.0%	4.6
	S6	60.7%	66.4%	5.7	71.3%	4.9
VR scenes	S7	64.6%	74.4%	9.8	81.6%	7.2
	S8	59.6%	72.5%	12.9	79.3%	6.8
	S9	62.7%	73.4%	10.7	80.5%	7.1

Table 2 shows that after training, the motor imagery recognition rate of the nine subjects was improved. In addition, the overall increase in the recognition rate in the static scenes (control group) and the dynamic scenes (experimental group I) was roughly the same, and the overall increase of the recognition rate in the VR scenes (experimental group II) was significantly higher than that of the other two groups. This result indicated that the rehabilitation training strategy designed in this study could improve the subjects’ EEG signal identification and ability to control EEG signals. Compared with static scenes and dynamic scenes, VR scenes had a more significant effect on improving subjects’ control of their motor nerves.

## Discussion

The experiments designed in this study compared the degree of neural activation as well as the classification and recognition rate of motor imagery when subjects performed motor imagery using stimulation with different virtual scenes. The rehabilitation training strategy designed in this paper greatly improved the motor nerve activation of the subjects, accelerated the remodeling of the subjects’ neural functions, and improved the motor imagery ability of the subjects.

The experiments further investigated in which scene the subjects’ motor nerve activation layers were wider and deeper.

The virtual scenes designed in this study included static scenes (e.g., pictures, text), dynamic scenes (e.g., animation, games) displayed on a computer screen, and VR scenes. After completing the motor imagery training, we compared and analyzed the energy changes in the brain motor areas and the motor imagery recognition rate changes for the different subjects to explore the neural activation and motor imagery ability changes of subjects using stimulation of different virtual scenes.

First, we analyzed the energy changes in the brain motor areas of subjects under different scene simulation and analyzed the BEAM of one subject randomly selected from each of the three groups. By comparing the distribution of BEAM of each subject before and after training, we found that the range of energy distribution in the motor area after training expanded and the color depth deepened. This proved that after training with the rehabilitation strategy described in this paper, the neural activity intensity and energy in the motor areas of subjects increased, which could stimulate the remodeling of damaged motor nerves to a certain extent.

We further analyzed motor imagery recognition rates. First, the recognition rates of online training (Table 1) and offline assessment (Table 2) of the nine subjects under different virtual scene simulation remained at around 60%. The main reason that the recognition rate of the latter was slightly higher than that of the former was that the signal interference was less formed in the offline state. Second, from the data in Table 1, we found that with an increase in the number of training days, the motor imagery recognition rate of the nine subjects followed an overall upward trend. The fluctuation of the recognition rate in the short period was affected mainly by the mental state of the subjects during the experiment and the interference differences of the EEG signals in each acquisition. Third, by calculating the average recognition rate of 14 days, we found that the recognition rate of subjects in the VR scenes was significantly higher than that of the static scenes and dynamic scenes displayed on a computer screen. This result showed that the subjects could mobilize more nerve cells in the motor area to pursue regular physiological activities in the VR scenes, thereby improving the recognition of the subjects’ EEG signals. As shown in Table 2, to verify whether the improved recognition rate could be formed by the transient stimulation of the virtual scenes or by the change of the motor nerve activity mechanism, we adopted the same experimental paradigm of motor imagery for offline assessment. We found that the improvement of the recognition rate in the VR scenes was significantly better than that in the static and the dynamic scenes, which was consistent with previous results, and further indicated that the VR scenes had a better effect on improving the recognition of subjects’ EEG signals.

## Conclusion

Today, the virtual rehabilitation that integrates MI-BCI and VR holds significant potential in the field of stroke rehabilitation, and this technology can greatly improve the rehabilitation effect for patients. To investigate how MI-BCI therapy can maximize the deep activation of subjects’ motor nerves and accelerate the remodeling mechanism of motor nerve function, we designed the following rehabilitation training strategy: we enhanced motor imagery training under different virtual scenes and compared and analyzed the degree of neural activation and the recognition rate of motor imagery in stroke patients after enhanced motor imagery training using stimulation with different virtual scenes. The experimental results showed that the motor imagery training using virtual scene stimulation could improve the motor nerve activation and motor imagery ability of the subjects. Compared with the static and dynamic scenes displayed on a computer screen, the VR scenes had a more significant effect in improving neural activation intensity and recognition rate.

## Data Availability Statement

The raw data supporting the conclusions of this article will be made available by the authors, without undue reservation.

## Ethics Statement

The studies involving human participants were reviewed and approved by the Human Ethics and Administrative Council of Yanshan University. The patients/participants provided their written informed consent to participate in this study.

## Author Contributions

PX, XC, and ZW designed this study. ZW wrote the manuscript. ZL, YW, NW, and ZL contributed to conception of the study. JW and XC wrote sections of the manuscript. All authors contributed to manuscript revision, and approved the submitted version.

## Conflict of Interest

The authors declare that the research was conducted in the absence of any commercial or financial relationships that could be construed as a potential conflict of interest.

## Publisher’s Note

All claims expressed in this article are solely those of the authors and do not necessarily represent those of their affiliated organizations, or those of the publisher, the editors and the reviewers. Any product that may be evaluated in this article, or claim that may be made by its manufacturer, is not guaranteed or endorsed by the publisher.
